# Bedtime procrastination related to loneliness among Chinese university students during post-pandemic period: a moderated chain mediation model

**DOI:** 10.1186/s12889-024-18019-6

**Published:** 2024-02-16

**Authors:** Cheng Xu, Nongying Lin, Zhiyu Shen, Zhaoyang Xie, Duo Xu, Jingdong Fu, Wenhua Yan

**Affiliations:** 1 School of Psychology and Cognitive Science, East China Normal University, Shanghai, China; 2 Shanghai Key Laboratory of Mental Health and Psychological Crisis Intervention, School of Psychology and Cognitive Science, East China Normal University, Shanghai, China 200062

**Keywords:** Post-pandemic period, Loneliness, Bedtime procrastination, Risk perception of COVID-19, Self-regulatory fatigue, Connectedness to nature

## Abstract

**Background:**

This study examined the relationship between loneliness and bedtime procrastination among Chinese university students, the mediating effects of COVID-19 risk perception and self-regulatory fatigue, and connectedness to nature’s protective role, post pandemic.

**Methods:**

We recruited 855 students to complete the Loneliness, Perceived Risk of COVID-19 Pandemic, Self-Regulatory Fatigue, Bedtime Procrastination, and Connectedness to Nature Scales. Data for descriptive statistics, correlation analysis, and moderated chain mediation effects were analyzed using SPSS 24.0 and process 3.5 macros.

**Results:**

Loneliness positively correlated with bedtime procrastination, COVID-19 risk perception mediated the impact of loneliness on bedtime procrastination, self-regulatory fatigue mediated the effect of loneliness on bedtime procrastination, and COVID-19 risk perception and self-regulatory fatigue mediated the effect between loneliness and bedtime procrastination. Furthermore, connectedness to nature mediated the impact of COVID-19 risk perception on self-regulatory fatigue.

**Conclusions:**

The results indicate the effects and potential mechanisms of loneliness on bedtime procrastination after the relaxation of the pandemic prevention and control policy in China from the perspective of self-regulatory resources and provide insights into improving university students’ sleep routine and mental health post pandemic.

## Introduction

The outbreak of COVID-19 not only causes tremendous public property damage and public safety hazards but also threatens the public's physical and mental health. Several studies have suggested that the combination of physical and psychological effects may lead to the emergence and exacerbation of sleep problems [1, 2], affecting the regularity of people's routines and disrupting sleep rhythms [3]. University students, as representatives of the young population, appear to be more vulnerable to psychological and post-traumatic stress during the outbreak of COVID-19, which in turn leads to sleep problems [4–6]. A recent survey conducted in North America showed that university students’ sleep–wake patterns changed during the pandemic [7]. Many have experienced multiple sleep problems [8], and bedtime procrastination has become more common. Similarly, the Chinese Sleep Research Society [9] published a white paper on sleep among Chinese residents during the 2020 pandemic lockdown, which found a significantly late bedtime, with more than 50% going to bed after midnight.

Recently, China entered a post-pandemic period. On December 7, 2022, based on the current pandemic situation and virus variations, China issued "10 new measures " [10] to optimize the COVID-19 response. The number of positive nucleic acid tests in the reported population increased continuously in all provinces, reaching a peak of 6.94 million on December 22 [11]. The risk of a pandemic remains high, and its negative consequences remain critical factors in individuals’ physical and mental health.

A review of previous studies revealed that most research on bedtime procrastination related to mental health focused on the COVID-19 pandemic [12, 13]. However, few studies have yet to be conducted during the post-pandemic period. Although the lockdown was over, the pandemic is ongoing and the negative effect of pandemic is also ongoing. A prediction published by Nature News on June 9, 2023 shows that China will usher in a peak of infections every 6 months [14]. The virus that causes COVID-19 is constantly changing over time. On Dec. 8, 2023, JN.1 is being shown separately for the first time on Centers for Disease Control and Prevention(CDC)’s SARS-CoV-2 Nowcast, and CDC predicts that COVID-19 activity is likely to increase [15]. At the same time, many research found that some individuals infected with COVID-19 had persistent or new symptoms that lasted several months beyond their initial infection, which influenced their life mentally and physically [16, 17]. Moreover, insomnia triggered by stressful life events continues for a long period [18]. After university students returned to campus, their wake-up time advanced and sleep debt increased [19], and they are the group that is most likely to have mental health problems during any public health emergency [20]. Focusing on loneliness and bedtime procrastination among university students during this period has practical implications for improving mental health and gains experience for similar public health issues in the future.

From a theoretical perspective, most scholars have examined the mechanisms of loneliness and bedtime procrastination from the perspective of coping with stress and physiological responses [21, 22]. However, in response to COVID-19, high levels of uncertainty and risk perception can lead to negative emotions and maladaptive behaviors [23–26], causing individuals to consume psychological resources to control their state [23, 27]. Thus, it may be possible to further explain the impact and underlying mechanisms of negative experiences (loneliness) on bedtime procrastination from a self-regulatory perspective. Ego depletion theory points out that psychological resources are indispensable to the executive function of the self, and psychological resources are limited, and psychological resources will be reduced after self-control [28]. The impact of emotions on psychological resources has always been one of the hot spots of research. When psychological resources are consumed, individual behavior and cognition will also change accordingly [29]. In the post-epidemic period, university students still have the same negative emotions and psychological problems as during the epidemic, which will consume individuals' psychological resources for adjustment and affect participants' perception of external risks and bedtime behavior in the process. This study will provide a theoretical basis for reducing emotional and behavioral problems in the post-pandemic period.

In addition, previous studies exploring the adverse effects of chronic stressors, such as COVID-19, on individuals have often considered the role of individual traits, such as personality traits, cognitive-emotional regulation, and coping styles [30–32], or the role of social environment variables, such as social support and parent–child relationships [33, 34], but often overlook the role of the natural environment. The consequences of psychological resource depletion are often negative, and it is necessary to explore ways to mitigate or even eliminate such negative consequences [29]. Some studies have suggested that natural exposure facilitates the replenishment of self-control resources [35], which is likely to have significant positive implications for an individual's psychological well-being [36]. Therefore, it is also important for this study to further explore whether connectedness to nature is a protective factor for the psychological condition of university students in the post-pandemic period and whether it can provide long-term positive suggestions for improving the emotional experience and sleep quality.

### Loneliness

Loneliness is a negative emotional experience that arises when individuals do not fulfill their interpersonal relationships [37]. Most people sometimes experience loneliness [38]. However, the beneficial aspects of loneliness, which is a universal but avoidable subjective experience, diminish when the emotions are intense and persistent, especially when individuals are trapped in long-term and chronic feelings of loneliness, which can negatively affect both physical and mental health [39, 40]. In addition, some individuals experience poor sleep quality throughout their lifespans [41, 42]. Today, an increasing number of people are beginning to report frequent, severe, and persistent feelings of loneliness [43, 44], and the situation has been worsened by COVID-19 [45]. People experience an overall increase in loneliness during the pandemic, such as older adults asked to self-isolate [46], parents who are unable to see their children [47], and adolescents whose social activities are reduced and academic lives are affected [48]. The restriction of social distancing and the anxiety of being infected under the lockdown brought a great deal of stress and loneliness among university students [49, 50]. It has also been suggested that the loneliness associated with COVID-19 leads to more intense negative emotions [51] and a range of sleep problems [52], which severely disrupt students' transitions to university and later school plans, causing numerous mental health problems [53]. With the relaxation of prevention and control policies in China, individuals are at an extremely high risk of infection or reinfection and are asked to be responsible for their health [54], so self-prevention remains necessary. Interpersonal interaction and campus life still influence university students [55]. Thus, the frequency of social activities is still low [56], and the impact of loneliness on individuals’ lives cannot be ignored.

### Bedtime procrastination

Procrastination refers to the voluntary delay of a desired course of action despite knowing that it would be worse to put it off [57]. Kroese et al. [58] introduced procrastination to the sleep domain for the first time, introducing the concept of "bedtime procrastination" to describe the phenomenon of people's inability to go to bed at their scheduled time without external environmental disturbances. The short-term mood repair of procrastination suggests that it can be viewed as a phenomenon in which short-term emotional processing takes precedence over long-term goal attainment [59]. Dealing with negative emotional states, such as loneliness, is often central to understanding the emergence of procrastination behaviors [60]. A similar perspective holds in the sleep domain. In addition to influencing bedtime state, negative emotions also cause procrastinators to exhibit negative expectations about the future and trigger bedtime procrastination [61, 62]. Therefore, this study hypothesized that loneliness in the post-pandemic university population positively correlates with bedtime procrastination.

### The mediating role of risk perception of COVID-19

Risk perception refers to an individual's reliance on intuition to estimate and judge risky events [63]. Previous studies have found that loneliness significantly influences risk perception. Loneliness affects the way a person views and thinks about the world and others [64] and is a risk factor for increased negative and depressive perceptions and increased sensitivity to threats [65], which may lead to changes in an individual's perception of risk. Cacioppo et al. [66] outlined the characteristics of lonely individuals from an evolutionary perspective, noting that loneliness leads to self-protective bias and invisible vigilance to threats. Thus, loneliness leads to higher threat perceptions [67], which can cause lonely individuals to make more negative assessments when faced with unexpected situations such as COVID-19 [68], interpret the current environment as threatening, and be in a state of chronic vigilance [21]. Loneliness has the potential to reinforce individuals' risk perceptions of COVID-19. Studies have shown a strong link between risk perception and stress [69, 70]. Implicit theories about willpower state that stress may lead to procrastination, applying to learning procrastination [71, 72] and bedtime procrastination [73]. Furthermore, bedtime procrastination is a short-term, active emotion regulation strategy used by individuals to adjust their emotions to a state suitable for sleep [74]; therefore, when individuals are in a stressful situation due to a high level of risk perception, they are likely to delay going to bed to help them cope with their negative emotions. Accordingly, the present study hypothesized that the risk perception of COVID-19 is an important mediating variable between loneliness and bedtime procrastination among university students during the post-pandemic period.

### The mediating role of self-regulatory fatigue

Self-regulatory fatigue is persistent fatigue arising from an individual's chronic depletion of self-control resources [75] must constantly regulate their cognition, emotions, and behavior daily by their social goals [76]. According to ego depletion theory, individuals have limited resources for self-control to cope with adverse situations and emotions [77, 78]. Each time a person transcends, suppresses, or changes negative emotions such as loneliness, it increases the depletion of self-regulatory resources, and the interpersonal interactions faced by lonely individuals tend to be more demanding, which can result in a more significant cognitive burden [78, 79]. Higher levels of loneliness are likely to lead to a more significant depletion of self-regulatory resources [79, 80]. Meanwhile, many researchers have identified self-regulation’s crucial role in bedtime procrastination [81, 82]. In the formal model of procrastination, similar to general procrastination, an individual’s self-control affects bedtime procrastination [83]. When excessive self-regulatory resources are consumed during the day, they are temporarily depleted, which leaves the organism in a state of self-exhaustion, leading to insufficient resources to perform regulation at night, failure of self-regulation, and bedtime procrastination at last [78, 84]. Therefore, we hypothesized that self-regulatory fatigue would mediate the relationship between loneliness and bedtime procrastination.

### The chain mediating role of risk perception of covid-19 and self-regulatory fatigue

Cameron et al. [85] integrated the common sense model [86, 87] and temporal self-regulation theory [88] to construct a framework for the self-regulation of disease risk, suggesting that risk representations trigger emotional responses such as fear and worry and that both risk itself and emotional arousal contribute to cognitive regulation efforts The risk perception of COVID-19 and self-regulatory fatigue might play a chain mediating role in the effect of loneliness on bedtime procrastination. During the post-pandemic period, individuals continue to perceive the risk of COVID-19, and the resulting negative emotions are likely to exacerbate the burden of self-regulation. Furthermore, the cognitive maps of risk attitudes and perceptions proposed by Slovic [63] argue that uncertainty is closely linked to individual risk perception [89], and extreme uncertainty resulting from COVID-19 is often accompanied by an individual overestimation of risk [90, 91]. In an environment of uncertainty, individuals tend to increase their sense of control to reduce uncertainty [91, 92], all of which challenges their ability to self-regulate [93].

### The moderating role of connectedness to nature

Connectedness to nature occurs when individuals experience a sense of connection with nature [94]. Hypervigilance to external risks requires limited self-control resources and may lead to increased cognitive load, leaving the individual in a state of self-depletion [65, 79]. In contrast, the restorative function of connectedness to nature can effectively replenish depleted resources [95] and improve self-control [96]. The theory of stress recovery during exposure to natural and urban environments suggests [97] that humans living in a natural environment for long periods can relieve their physical and mental stress through the natural environment, where the stress-reducing effect of connectedness to nature is mainly because of the restoration of self-control resources. This suggests that connectedness to nature facilitates the filling of resource gaps, thus relieving fatigue and producing positive effects in areas such as cognition [98]. Higher perceptions of pandemic risk can put individuals in a state of stress [99], bring about negative emotions [100], and trigger more significant uncertainty [101], all of which can lead to limited cognitive resources [65]. In contrast, connectedness to nature can alleviate the state of alertness and restore resources. Therefore, it is likely to compensate for the adverse effects caused by the risk perception of COVID-19 and mitigate the adverse effects. Based on this, the present study verified whether connectedness to nature could act as a protective factor to mitigate the effects of the risk perception of COVID-19 on self-regulatory fatigue.

**Fig. 1:**
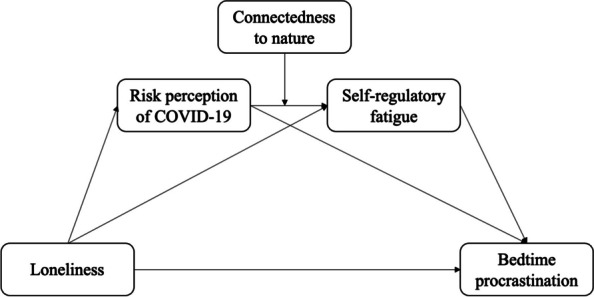
Proposed model

In summary, this study constructs a moderated chain mediation model using the following hypotheses (Fig. 1):

- H1: Loneliness positively correlates with bedtime procrastination.
- H2: Risk perception of COVID-19 mediates the effect of loneliness on bedtime procrastination.
- H3: Self-regulatory fatigue mediates the effect of loneliness on bedtime procrastination.
- H4: Risk perception of COVID-19 and self-regulatory fatigue play a chain mediating role in the effect of loneliness on bedtime procrastination.
- H5: Connectedness to nature moderates the effect of the risk perception of COVID-19 on self-regulatory fatigue.

## Methods

### Participants

This study used convenience sampling to select university students from January to February 2023, following the change in China's epidemic policy. We distributed 993 questionnaires. One hundred and thirty-eight invalid (inattentive, missing, regular responses) questionnaires were excluded; finally, 855 valid questionnaires were obtained, with an effective rate of 86.10%. Among them, 460 were male (53.80%), and 395 were female (46.20%). Participants were between 18 and 32 years old (*M* = 21.16; *SD* = 1.83). In this study, 508 (59.40%) university students came from cities in China, 247 (28.90%) came from rural areas in China, and 100 (11.70%) came from towns. Among the participants, 133(15.60%) were junior college students, 667 (78.00%) were undergraduate students, and the other 55 (6.50%) were graduate and doctoral students. In this study, 703 (82.22%) reported being infected with COVID-19 in the last three months and 759 (88.77%) participants reported that their loved ones and friends had been infected with COVID-19 in the last three months. This study was conducted with the approval of the Ethics Committee of East China Normal University (No: HR1-0130–2022).

### Instruments

#### Loneliness scale

The Loneliness Scale [102, 103], revised by Wang, was used. There are 18 items on the scale, such as "I feel I lack the friendships of others", and "I do not feel lonely ". The scale is rated on a 4-point Likert scale ranging from 1 (never) to 4 (always). The higher the total score, the greater the sense of loneliness. Questions 1, 4, 5, 6, 8, 9, 14, 15, 16, and 18 are reverse scored. This scale applies to Chinese university students and is reliable [103]. A confirmatory factor analysis showed that construct validity of the scale was acceptable (CMIN/DF = 6.19, RMSEA = 0.08, CFI = 0.91, TLI = 0.87). The standardized factor loadings for all items were between 0.30–0.82. In this study, the Cronbach’s alpha for the questionnaire was 0.90.

### Perceived Risk of COVID-19 Pandemic Scale (PRCPS)

The Perceived Risk of the COVID-19 Pandemic Scale (PRCPS) developed by Xi et al. was used [104]. There are nine items in the scale, such as "How likely do I think I am to get COVID-19", and "I am likely to get COVID-19 no matter how small the chance is". A Liker scale of 4–6 was used. The higher the total score, the higher the risk perception of COVID-19, with one question "I am sure I will not get COVID-19" being reverse scored. This scale is applicable to all age groups in China and has good reliability [104]. A confirmatory factor analysis showed that the scale had good construct validity (CMIN/DF = 2.47, RMSEA = 0.04, CFI = 0.99, TLI = 0.98). The standardized factor loadings for all items were between 0.33–0.82. In this study, Cronbach’s alpha for the questionnaire was 0.80.

### Self-Regulatory Fatigue Scale (SRF-S)

The Self-Regulatory Fatigue Scale (SRF-S) developed by Nes et al. and modified by Wang et al. [105, 106] was used to assess the level of self-regulatory fatigue. The scale has 18 items, such as "I feel energetic", and "I have difficulty executing my exercise program". A 5-point Likert scale ranging from 1 (strongly disagree) to 5 (strongly agree) was used. The higher the total score, the greater the degree of self-regulatory fatigue. Questions 1, 2, 5, 9, 11 and 14 were reverse-scored. This scale is reliable and applicable to the Chinese youth population and has good reliability [106]. Considering that the factor loading of question 14 is less than 0.3, in addition, the residual correlation between question 5 and question 13, question 1 and question 3, question 4 and question 9 is high, so the questions with low factor loading among questions with high residual correlation are deleted. Questions 14, 13, 3 and 9 was deleted [107]. After deletion, the number of questions in each dimension is still greater than 3. We conducted confirmatory factor analysis on the remaining questions, and the results showed that construct validity of the scale was good (CMIN/DF = 5.8, RMSEA = 0.08, CFI = 0.95, TLI = 0.93). The standardized factor loadings for all items were between 0.60–0.80. In this study, Cronbach’s alpha was 0.88.

### Bedtime Procrastination Scale (BPS)

The Bedtime Procrastination Scale (BPS) developed by Kroese et al. and modified by Ma et al. [58, 108] was used. There are nine items on the scale, such as "I go to bed later than expected", and "I easily stop the activity I am doing if it is time to go to bed". A 5-point Likert scale ranging from 1 (rarely) to 5 (almost always) was used. The higher the total score, the more severe the bedtime procrastination. Questions 2, 3, 7, and 9 were reverse-scored. This scale is reliable for Chinese university students [108]. A confirmatory factor analysis showed that the scale had good construct validity (CMIN/DF = 3.31, RMSEA = 0.05, CFI = 0.99, TLI = 0.98). The standardized factor loadings for all items were between 0.38–0.85. In this study, Cronbach’s alpha was 0.89.

### Connectedness to Nature Scale (CNS)

The Connectedness to Nature Scale (CNS) developed by Mayer et al. and revised by Na et al. [94, 109] was used. The scale has 14 items, such as "I often feel at one with nature," and "My well-being is not related to the good or bad aspects of nature". A 5-point Likert scale ranging from 1 (strongly disagree) to 5 (strongly agree) was used. The higher the total score, the stronger the individual's connectedness to nature. Questions 4, 12, and 14 were reverse-scored. This scale is reliable and applicable to Chinese university students and has good reliability [109]. A confirmatory factor analysis showed the standardized factor loadings of question 4, 12, 13 and 14 were lower than 0.3. Therefore we delete these questions. For the remaining 10 items we conducted confirmatory factor analysis and results showed that the scale had good construct validity (CMIN/DF = 3.66, RMSEA = 0.06, CFI = 0.96, TLI = 0.94). In this study, Cronbach’s alpha for the questionnaire was 0.83.

### Statistical analysis

SPSS 24. 0 and process 3.5 macro programs were used to perform descriptive statistical analysis, correlation analysis, and moderated chain-mediated effect tests on the data. Confirmatory factor analysis was performed using Mplus 8.0.

## Results

### Common method deviation test

As the results may be affected by common method bias owing to the use of self-reported methods for data collection, the Harman one-way test was used to test for common method bias [110]. Fifteen common factors with eigenvalues greater than one were obtained without rotation, with the first common factor having an explanatory rate of 21.16%, well below 40%. No serious common method bias was observed.

### Descriptive statistics and correlation analysis among variables

Table 1 presents the results of the descriptive statistics and correlation analyses for each study variable. Significant positive correlations were found between loneliness, risk perception of COVID-19, self-regulatory, and bedtime procrastination.

**Table 1:** Descriptive statistics and correlations among main variables

Variable	*M*	*SD*	*1*	*2*	*3*	*4*
1 Loneliness	34.98	9.06	—			
2 Risk perception of COVID-19	32.17	5.62	0.13^**^	—		
3 Self-regulatory fatigue	29.56	9.02	0.76^**^	0.20^**^	—	
4 Bedtime procrastination	28.21	7.86	0.50^**^	0.20^**^	0.57^**^	—
5 Connectedness to nature	42.84	4.91	-0.39^**^	0.02	-0.35^**^	-0.22^**^

*N* = 855.^**^p < .01

In this study, 703 (82.22%) reported being infected with COVID-19 in the last three months. Independent sample t-tests found that this group of participants had higher levels of loneliness (*t* = 2.27, *p* < 0.05), risk perception of COVID-19 (*t* = 10.11, *p* < 0.001), self-regulatory fatigue (*t* = 2.85, *p* < 0.01), and bedtime procrastination (*t* = 3.38, *p* < 0.001). In this study, 759 (88.77%) participants reported that their loved ones and friends had been infected with COVID-19 in the last three months. Independent sample t-tests revealed higher levels of risk perception of COVID-19 (*t* = 5.00, *p* < 0.001) and bedtime procrastination (*t* = 3.11, *p* < 0.01) in this group of participants.

Model 6 in the macro process program developed by Hayes was used to test the mediating role of risk perception of COVID-19 and self-regulatory fatigue between loneliness and bedtime procrastination [111]. Gender and age were included as control variables. In addition, the effect of infection with COVID-19 on oneself, loved ones, and friends in the last three months on the study variables was considered. Therefore, the presence or absence of COVID-19 infection in oneself, loved ones, and friends over the last three months was also included as a control variable in the model.

The results (Table 2) showed that loneliness significantly and positively predicted bedtime procrastination (*β* = 0.50, *p* < 0.001). After including risk perception of COVID-19 and self-regulatory fatigue in the model, loneliness significantly and positively predicted risk perception of COVID-19 (*β* = 0.11, *p* < 0.001) and self-regulatory fatigue (*β* = 0.74, *p* < 0.001); risk perception of COVID-19 significantly and positively predicted self-regulatory fatigue (*β* = 0.10, *p* < 0.001) and bedtime procrastination (*β* = 0.09, *p* < 0.05); self-regulatory fatigue significantly and positively predicted bedtime procrastination (*β* = 0.43, *p* < 0.001); at this point, loneliness remained a significant predictor of bedtime procrastination (*β* = 0.16, *p* < 0.001).

**Table 2:** Test for the chain mediation model

Regression equation		Overall fit indices			Significance of regression coefficients		
Result Variables	Predictive variables	*R*	*R*^*2*^	*F*	*β*	*95%CI*	*t*
Bedtime procrastination	Loneliness	0.52	0.27	62.20^***^	0.50	[0.44,0.55]	16.78^***^
Risk perception of COVID-19	Loneliness	0.35	0.13	24.31^***^	0.11	[0.04,0.17]	3.32^***^
Self-regulatory fatigue	Loneliness	0.77	0.59	204.46^***^	0.74	[0.70,0.79]	33.44^***^
	Risk perception of COVID-19				0.10	[0.06,0.15]	4.46^***^
Bedtime procrastination	Loneliness	0.60	0.36	67.32^***^	0.16	[0.08,0.25]	3.86^***^
	Risk perception of COVID-19				0.09	[0.03,0.14]	2.85^**^
	Self-regulatory fatigue				0.43	[0.34,0.51]	9.92^***^

*N* = 855, ^*^p < 0.05, ^**^p < 0.01,^***^p < 0.001. In addition to age and gender, the presence or absence of COVID-19 infection in oneself, loved ones, and friends over the last three months were also included as a control variable in the model. The study variables were standardized

The results of the mediation effect analysis (see Table 3) showed that the mediated effect value of risk perception of COVID-19 was 0.009, that the mediated effect value of self-regulatory fatigue was 0.318, and that the effective value of the chain mediated effect of risk perception of COVID-19 and self-regulatory fatigue was 0.005. The bootstrap 95% confidence intervals for all three mediated paths did not contain zero, and all three mediated effects reached significant levels, accounting for total effects of 1.82%, 64.24%, and 1.01%. Hypotheses 2, 3, and 4 are supported.

**Table 3:** Mediating effect analysis of the chain mediating model

	*Effect*	*SE*	*95%CI*
Total effect	0.495	0.030	[0.438, 0.553]
Direct effect	0.164	0.043	[0.081, 0.247]
Mediating effect of risk perception of COVID-19	0.009	0.004	[0.002, 0.019]
Mediating effect of self-regulatory fatigue	0.318	0.036	[0.250, 0.389]
Chain mediating effect of risk perception of COVID-19 and self-regulatory fatigue	0.005	0.002	[0.002, 0.009]

SE and 95% CI refer to the standard errors, lower and upper 95% confidence intervals of the indirect effects estimated by the bias-corrected percentile Bootstrap method, respectively

To explore the moderating role of connectedness to nature, Model 91 of the process macro program developed by Hayes was used [111]. The results (see Table 4) showed that after controlling for the variables such as age and gender, and whether getting infection of COVID-19 for oneself and loved ones and friends in the last 3 months, the coefficients of each path of the model reached significant levels, and the interaction of risk perception of COVID-19 and connectedness to nature had a significant predictive effect on self-regulatory fatigue (*β* = -0.06, *p* = 0.0094). This suggests that connectedness to nature moderates the effect of risk perception of COVID-19 on self-regulatory fatigue, and therefore, hypothesis 5 is supported.

**Table 4:** Test for the moderated chain mediation model

Regression equation		Overall fit indices			Significance of the regression coefficients		
Outcome variables	Predictors	*R*	*R*^*2*^	*F*	*β*	*95% CI*	*t*
Risk perception of COVID-19	Loneliness	0.35	0.13	24.31^***^	0.10	[0.04,0.17]	3.32^***^
Self-regulatory fatigue	Loneliness	0.77	0.60	158.14^***^	0.71	[0.66,0.76]	29.61^***^
	Risk perception of COVID-19				0.11	[0.06,0.16]	4.69^***^
	Connectedness to nature				-0.08	[-0.12,-0.03]	-3.21^***^
	Risk perception of COVID-19* Connectedness to nature				-0.06	[-0.10,-0.01]	-2.60^**^
Bedtime procrastination	Loneliness	0.60	0.36	67.32^***^	0.16	[0.08,0.25]	3.86^***^
	Risk perception of COVID-19				0.09	[0.03,0.14]	2.85^*^
	Self-regulatory fatigue				0.43	[0.34,0.51]	9.92^***^

*N* = 855, ^*^p < 0.05, ^**^p < 0.01,^***^p < 0.001. In addition to age and gender, the presence or absence of COVID-19 infection in oneself, loved ones, and friends over the last three months were also included as a control variable in the model. The study variables were standardized

**Fig. 2:**
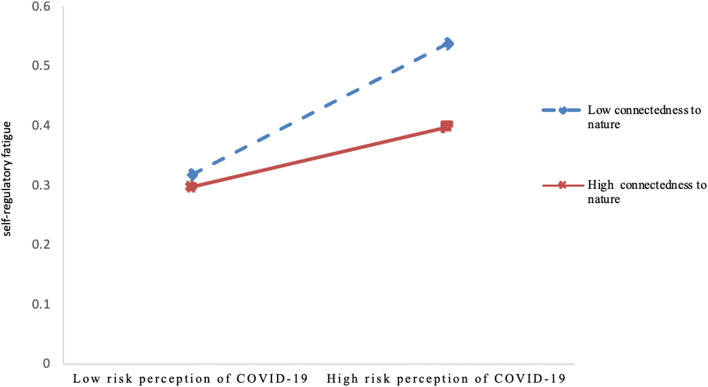
The moderating role of connectedness to nature between risk perception of COVID-19 and self-regulatory fatigue

We performed a simple slope analysis better to illustrate the moderating role of connectedness to nature. Figure 2 shows that for participants with low connectedness to nature (*M-SD*), the risk perception of COVID-19 significantly and positively predicted self-regulatory fatigue (*simple slope* = 0.17, *p* < 0.001), whereas, for participants with high connectedness to nature (*M* + *SD*), the risk perception of COVID-19 was not a significant predictor of self-regulatory fatigue (*simple slope* = 0.05, *p* = 0.111). This suggests that the effect of the risk perception of COVID-19 on self-regulatory fatigue decreases significantly as connectedness to nature increases.

## Discussion

From the perspective of self-regulatory resources, this study is based on the pandemic situation in the new era, the "limited" nature of individual psychological resources, and the characteristics of university students' high psychological stress and emotional problems. By constructing a moderated chain mediation model, we investigated the effects of loneliness on university students' bedtime procrastination and the underlying psychological mechanisms. The results showed that, in the post-pandemic period, university students' loneliness correlated with bedtime procrastination through both direct and indirect pathways. In addition, connectedness to nature moderated the effect of risk perception of COVID-19 on self-regulatory fatigue.

### The relationship between loneliness and bedtime procrastination

This study found that loneliness significantly and positively correlated with bedtime procrastination, consistent with previous findings [52, 112]. University students, as a target group among young people, have been shown to experience widespread loneliness in several studies [113, 114]. A short-term focus on adjusting to negative experiences and feelings associated with negative emotions, such as loneliness, can lead to a failure of control in other areas of life [115], such as an increase in bedtime procrastination. This negative expectations to the future causes individuals to delay bedtime to slow the arrival of the next day [62].

### The mediating role of risk perception of covid-19

In a post-pandemic environment, loneliness caused by a lack of social bonds triggers more negative perceptions, making individuals tend to interpret environments as having high risk [21, 65], and suffer higher levels of distress. Risk perception positively predicts individual stress levels [116], which can further exacerbate a range of negative emotions[117]. Based on emotional responses to the risk perception of COVID-19, uncertainty arises and leads to multiple types of maladaptive problems [118], ultimately affecting bedtime procrastination [119].

### The mediating role of self-regulatory fatigue

The present study also confirmed that loneliness could correlated with bedtime procrastination in university students through the mediating role of self-regulatory fatigue, which further supports ego depletion theory [77, 78]. When an individual's limited resources are greatly depleted, the individual enters a state of ego depletion, leading to bedtime procrastination. Additionally, loneliness motivates individuals to regulate their emotions and seek closer social connections [65, 120]. However, during the adjustment process, both the negative emotions themselves and the demanding interpersonal interactions in the pandemic environment increase the burden of cognitive processing [78, 79], thus consuming more psychological resources and making individuals more likely to fall into a state of self-regulatory fatigue and delay bedtime [84].

### The chain mediating role of risk perception of COVID-19 and self-regulatory fatigue

In addition, the findings showed significant chain mediating effects of risk perception of COVID-19 and self-regulatory fatigue. Loneliness can make individuals more susceptible to perceptions of insecurity in the social environment and heightened sensitivity to external risks [69, 112], while elevated perceptions of pandemic-related threats can generate stress and negative emotions, which in turn deplete intrinsic attention and cognitive resources, lead to de-inhibition and self-regulatory fatigue, and trigger bedtime procrastination [93, 121].

Moreover, elevated risk perceptions due to loneliness can lead to higher uncertainty. The information-seeking and processing model of risk suggests that individuals at higher risk tend to actively gather risk-related information to construct defensive attitudes, beliefs, and behaviors to maintain their health due to uncertainty about risky events [122]. Therefore, to cope with the high-risk perception of COVID-19 and uncertainty in the current post-pandemic period, university students, who are the main users of the Internet medium [123], are more inclined to seek more knowledge and information through the Internet to reduce the sense of threat and negative emotions caused by uncertainty [118, 124, 125]. However, various online platforms and related reports are filled with a large amount of information that is difficult to distinguish between true and false and often overwhelms individuals, causing information overload, taking up more limited cognitive resources [126], and leading to self-depletion.

### The moderating role of connectedness to nature

Our results also revealed that connectedness to nature mitigated the adverse effects of risk perception COVID-19 on self-regulatory fatigue. This result further supports the stress recovery theory during exposure to natural and urban environments [97]. Exposure to nature helps reduce fatigue, replenish self-control resources, and increase the availability of psychological resources in the face of the pandemic risk, allowing people to better cope with stressful situations [96, 127] and avoid ego-depletion states [95]. By contrast, individuals with low connectedness to nature have fewer psychological resources. Their over-perception of pandemic risk results in a cognitive load that is difficult to regulate [65, 79], leading them to become more susceptible to a state of self-regulatory fatigue and ultimately increase bedtime procrastination [128, 129].

### Implications and future directions

In contrast to previous perspectives on coping with stress and physiological responses, this study explains the relationship between loneliness and bedtime procrastination in the post-pandemic period from the perspective of regulatory resources, emphasizing the importance of psychological resources in regulating emotional experiences and daily routines. Considering the negative effect of COVID-19 pandemic is ongoing and it has brought with significant health, economic and social uncertainties, people will still face the challenge of self-control. Plus, From a future perspective, people are still facing the continued mutation of coronaviruses and may encounter similar public health crises in the future. COVID-19 has made considerable physical and psychological impacts on governments and individuals [130, 131]. Such impacts have been shown to continually affect individuals' daily lives and even influence their responses to future public health crises [131]. Therefore, this study provides us with a new perspective, that is, from the perspective of regulatory resources, we should understand the internal mechanism of how loneliness affects the sleep behavior of college students in the context of the current social environment which is full of uncertainty, which also provides more insights for evidence-based interventions.

Moreover, for university students, contact with nature is closely linked to daily travel and campus life and is an easy, convenient, and feasible measure to regulate mood. With the complete relaxation of pandemic prevention and control measures, university students' opportunities and frequency of going outside will gradually rebound, and their exposure to the natural environment will increase. The results of this study also call for university students to be more exposed to the natural environment, which may bring many physical and mental benefits.

However, the present study has some limitations. First, the data in this study were obtained from participants’ self-reports, and a possible subjective reporting bias may have affected the reliability of the results. Second, a cross-sectional study design was used in the study design, which made it difficult to reveal the causal relationships between the variables. Moreover, since the pandemic outbreak, China has taken strict preventive and control measures and insisted on "dynamic zero,” In contrast, the post-pandemic period, all the strict preventive and control measures for the pandemic were phased out in the short term. As people cope with high infection rates and risks, there is a greater need to adapt to sudden changes in policy and lifestyle, all of which can impact the individuals’ psychological state. Therefore, the post-pandemic period in China was unique. There may be differences in emotions and psychological adjustment resources between Chinese individuals and individuals in other countries post-pandemic, and future research could further examine research models in a cross-cultural scenario.

## Conclusions

This study explored the effects and potential mechanisms of loneliness on bedtime procrastination after the relaxation of the pandemic prevention and control policy in China from a new perspective. Results showed that loneliness significantly correlated with bedtime procrastination. Risk perception of COVID-19 mediated the effect between loneliness and bedtime procrastination. Self-regulatory fatigue also mediated the effect of loneliness on bedtime procrastination. Risk perception of COVID-19 and self-regulatory fatigue mediated the chain between loneliness and bedtime procrastination. Connectedness to nature mediates the effect of risk perception of COVID-19 on self-regulatory fatigue.

## Data Availability

The datasets used and/or analysed during the current study are available from the corresponding author on reasonable request.
